# Operant conditioning of stochastic chemical reaction networks

**DOI:** 10.1371/journal.pcbi.1010676

**Published:** 2022-11-18

**Authors:** David Arredondo, Matthew R. Lakin

**Affiliations:** 1 Center for Biomedical Engineering, University of New Mexico, Albuquerque, New Mexico, United States of America; 2 Department of Computer Science, University of New Mexico, Albuquerque, New Mexico, United States of America; 3 Department of Chemical & Biological Engineering, University of New Mexico, Albuquerque, New Mexico, United States of America; University of Pittsburgh, UNITED STATES

## Abstract

Adapting one’s behavior to environmental conditions and past experience is a key trait of living systems. In the biological world, there is evidence for adaptive behaviors such as learning even in naturally occurring, non-neural, single-celled organisms. In the bioengineered world, advances in synthetic cell engineering and biorobotics have created the possibility of implementing lifelike systems engineered from the bottom up. This will require the development of programmable control circuitry for such biomimetic systems that is capable of realizing such non-trivial and adaptive behavior, including modification of subsequent behavior in response to environmental feedback. To this end, we report the design of novel stochastic chemical reaction networks capable of probabilistic decision-making in response to stimuli. We show that a simple chemical reaction network motif can be tuned to produce arbitrary decision probabilities when choosing between two or more responses to a stimulus signal. We further show that simple feedback mechanisms from the environment can modify these probabilities over time, enabling the system to adapt its behavior dynamically in response to positive or negative reinforcement based on its decisions. This system thus acts as a form of operant conditioning of the chemical circuit, in the sense that feedback provided based on decisions taken by the circuit form the basis of the learning process. Our work thus demonstrates that simple chemical systems can be used to implement lifelike behavior in engineered biomimetic systems.

## Author summary

Programming the biological world in a rational manner is a key goal in synthetic biology and synthetic cell research. While much current work focuses on the practical tasks of realizing the fundamental biochemical processes of life in an engineered system, there is also the need to realize control circuitry that regulates the processes of life and enables adaptive behavior. This work builds toward this goal by designing chemical systems capable of probabilistic decision-making and adaptive behavior in stochastic reaction networks with species populations comparable to those found in naturally occurring single-celled microorganisms. By showing that these networks can exhibit non-trivial responses to their environment this work opens the door to lifelike behaviors in engineered synthetic cells.

## Introduction

Naturally occurring single-celled microorganisms exhibit a range of fascinating behaviors that arise from internal information processing. These range from chemotaxis in bacteria such as *E. coli* [1] to sophisticated decision-making avoidance responses to stimuli observed in single-celled eukaryotes such as *Stentor roeseli* [2]. The latter is of particular interest because recent work [3] found that the decision-making process of *Stentor* can be modeled as a random choice whether or not the animal should detach from its anchorage and swim to a new location to escape the stimulus. Further analysis of the results in that work has suggested that the animals may “learn” over time [4]. In either case, single-celled microorganisms make concrete decisions, often with an element of randomness, in response to environmental stimuli. Randomness presumably lends them an air of unpredictability that may offer survival advantages in the wild. It also lets the organism sample different outcomes of its choices, from which it may learn. Here, we explore a possible basis for probabilistic decision-making in chemical systems, opening the door to biomimicry in engineered biomolecular systems [5] or synthetic cells [6, 7].

**Fig 1:**
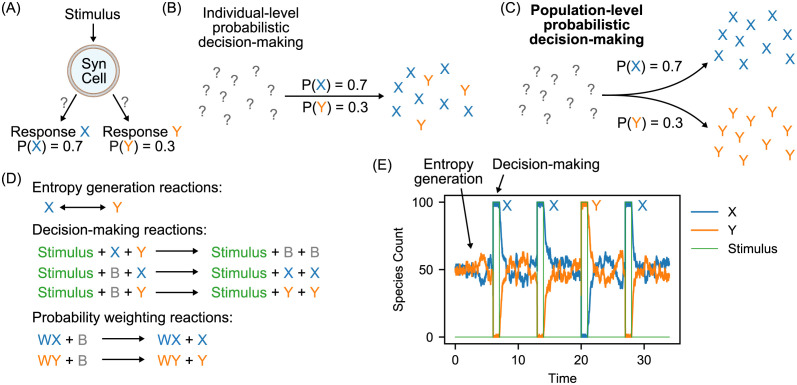
Probabilistic decision-making in stochastic chemical reaction networks. (A) Example application of probabilistic decision-making to drive the actions of a synthetic cell in response to external stimuli, via the random choice between one or more responses with particular probabilities. (B) Randomized decision-making at the level of individual chemical species produces roughly deterministic results when considered at the population scale, with decision molecules generated in proportion to the overall probabilities. (C) Here we propose a system for probabilistic decision-making at the population level, in which the entire population makes a coordinated transition into one decision state or the other based on the decision probabilities. (D) Basic design for probabilistic decision-making system. A simple reversible reaction between the X and Y species introduces entropy into the system. Then, when the Stimulus species is introduced, the approximate majority reaction system is activated, which converts all of the decision species into whichever happens to be in the majority when the stimulus is activated. This produces a randomized decision at the population level. To enable tuning of decision probabilities, additional weight species WX and WY can also recruit the “undecided” species B from the approximate majority reaction system to the corresponding decision species. The forward and backward rate constants for the entropy generation reactions are both *kNoise* = 1. The rate constants for the decision-making reactions are *kAM* = 1. The rate constants for the probability weighting reactions are *kBias* = 30. (E) Example stochastic simulation showing several sequence decisions between X and Y activated by the Stimulus species. In this example, the counts of the weighting species are WX=60 and WY=40, which translates to decision probabilities of P(X)=0.7 and P(Y)=0.3 (see Fig 2 for calculations that derive this translation).

Our approach is grounded in the field of molecular programming, which has made great strides in implementing information processing capabilities at the nanoscale using engineered biomolecules, primarily DNA. The experimental framework of toehold-mediated DNA strand displacement reactions [8] has enabled a wide range of computational systems to be realized in this manner, including digital logic circuits [9], distributed algorithms [10], and artificial neural networks [11, 12]. Theoretical work has shown that any abstract chemical reaction network (CRN), that is, a collection of reaction rules defining conversions between chemical species denoted as abstract mathematical objects, can be converted into a wet chemistry implementation using strand displacement [13]. Here, we study chemical reaction networks at this more abstract level, with the goal of demonstrating non-trivial behavioral phenotypes in this system that could subsequently be realized in the laboratory and used to control engineered synthetic cells or biorobots, as outlined in Fig 1A.

Chemical systems are inherently competitive and therefore lend themselves somewhat naturally to implementing probabilistic systems. In this work, we implement probabilistic decision-making in an abstract CRN, producing a system that can make probabilistic decisions in response to a stimulus at the *population* level. This is novel because much previous work in this direction has focused either on probabilistic decision-making at the single molecule level [14], which indirectly produces deterministic behavior at the population level, as shown in Fig 1B, or on computing probabilities without acting on them in a probabilistic manner [15, 16]. However, in our scheme a population of 100 individual molecules with 70:30 probabilities programmed for a decision between two possible responses X and Y to a stimulus, will either transition to 100 copies of the response X molecule with 70% probability or to 100 copies of the response Y molecule with 30% probability, as shown in Fig 1C, as opposed to simply producing a 70:30 split. Thus, the whole system commits to one of the possible responses, with probabilities that can be trained dynamically, as outlined below.

The specific goal of this work is to demonstrate *operant conditioning* of our simulated abstract CRNs. Operant conditioning is a form of conditioning popularized by Skinner [17] in his work on mice, in which animals are conditioned to carry out certain actions based on feedback given on previous actions in response to stimuli. This contrasts with classical conditioning, also known as associative learning [18], in which associations between two external stimuli are learned. Operant conditioning also bears some resemblances to reinforcement learning approaches used in mainstream machine learning research [19], in which agents receive rewards based on their actions. This task is particularly well-suited to our probabilistic decision-making framework because it requires the entire system to commit to a particular response before feedback is provided; given the decision-making system outlined below, this will occur with very high likelihood. Here we characterize a simple version of operant conditioning that uses a very simple reinforcement schedule of one feedback signal based solely on the most recent stimulus-response pair, and we discuss the possibility of implementing more sophisticated conditioning regimes in the Discussion below. Importantly, our systems operate in a population range on the order of 100–1000 copies of individual species, which is on the order of the numbers of copies of key molecules such as mRNAs within single-celled prokaryotic organisms. Our work demonstrates that decisions of this kind could be taken in a consistent manner and synchronized throughout the extent of a primitive synthetic cell, thereby providing new capabilities for the design and implementation of biomolecular systems capable of lifelike behaviors. Thus, while our approach is grounded in the theory of abstract chemical reaction networks, similar systems could in principle be used to implement adaptive decision-making behavior in rationally engineered synthetic cells, thus creating synthetic mimics that could be a valuable platform for exploring the molecular basis of such behaviors as witnessed in microorganisms by some of the earliest experimenters in the field of microbiology [2].

## Results

### Probabilistic decision-making

Our probabilistic decision-making CRN motif is based on the approximate majority (AM) scheme for achieving consensus in distributed systems; for a system with *n* agents initially composed of two competing types [20]. That system converts all members of a mixed population (X and Y, say) into whichever version is initially present in the majority, and has been shown to do so with very high probability if the initial margin is large enough (formally, $\Omega (\sqrt{n\;\text{log}\;n})$ [21]). It does so via an intermediate species B: an X and a Y combine to produce two B molecules and these are then catalytically recruited back to either X or Y; if one of X or Y is in the majority then a B is more likely to be recruited to the majority side, until none of the minority side remain. The AM system can be straightforwardly formulated as a CRN and indeed has been demonstrated in wet chemistry via a DNA strand displacement implementation [10] and closed-form solutions have been found for the AM system in the deterministic limit [22]. For our application, however, we want to be able to switch the entire population of molecules in a stochastic system either into state X or Y, with probability 0.5 for either choice in the first instance. Therefore, we introduce a novel element into the AM system: an *entropy generation module* that uses a simple reversible reaction between X and Y so that the species present in the majority becomes a random variable. In a stochastic system with equal rate constants for both sides of this reversible reaction, the counts of X and Y fluctuate around an equilibrium value which will be at a 1:1 ratio of X and Y. Then, we use an external stimulus signal to activate the AM reactions at a particular point, which causes the AM species to collectively switch either to all X or all Y based on which happened to be in the majority at that point in time. This produces a 50:50 probability of each choice being made. Removing the stimulus removes the drive to remain in that state and the reversible reaction causes the system to collapse back into a noisy state around the 1:1 equilibrium point. Thus, multiple such choices can be made sequentially given repeated applications and removals of the stimulus. The chemical reactions that implement this system are presented in Fig 1D and an example timecourse of multiple such choices is shown in Fig 1E, for a system containing 100 individuals. This demonstrates our basic system for coordinated probabilistic decision-making throughout a multi-component chemical system. The basic system consists of two modules: entropy generation and decision-making, each with its own rate constant, *kNoise* and *kAM*, respectively. While the stimulus-catalyzed approximate majority reactions push the system toward a consensus of state species, the entropy generation module creates noise in the consensus, so the rate *kAM* should be sufficiently high so as to maintain a dominant state species while stimulus is present. The higher the rate *kNoise*, the more quickly the system will return to an average 1:1 ratio of state species in the absence of stimulus, erasing the system’s memory of the previous state.

### Tuning probabilities

**Fig 2:**
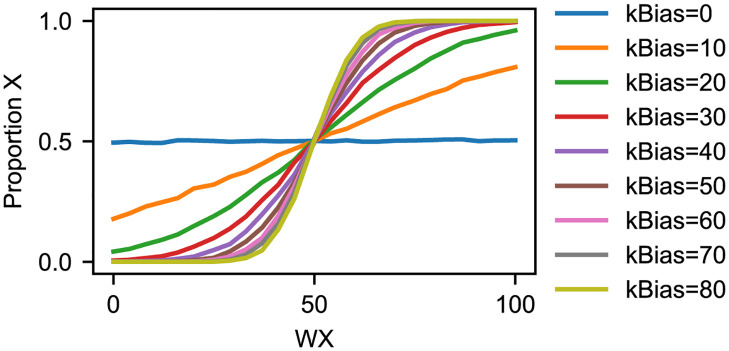
Analysis of decision probabilities as a function of weighting species counts. In each of 10^5^ traces, a CRN is initialized with 50 of each state species X and Y, and variable amounts of the weight species WX (plotted on x-axis) and WY, such that WX+WY=100. Each trace is 0.5 time units. After 0.45 time units, 100 molecules of the Stimulus species are added, and the state chosen is recorded, where a state is defined by 90 or more state molecules when sampled at *t* = 0.475 (halfway between the Stimulus perturbation and the end of the simulation). The proportion of traces that resulted in the X state is shown on the y-axis. The rate *kBias* is varied, and the common parameters are as follows: *kAM* = 1, *kNoise* = 1.

Our next step was to implement decision-making probabilities beyond 50:50, that is, equal probabilities of both choices being made. We therefore generalized our system into a weighted variant of the AM scheme which uses additional “weight” molecules as catalysts to bias the system into one state or the other [23]. The probability weighting module acts only on the B species, driving the system to consensus more quickly. The weight species act exactly as the state-species in converting B to their respective states, but are not degraded themselves when interacting with each other as the state species do. They thus provide a permanent catalyst to bias the recruitment of the undecided B species. The presence of such weights can be used to force the system to select the species initially in the minority rather than the majority, which in previous work on AM systems has been interpreted as making a “wrong” choice, and something to be avoided. Here, however, we treat this as a feature which allows us to achieve decision probabilities other than 50:50 by weighting the system such that the species present in the minority at the point when the stimulus is activated can sometimes win out. The chemical reactions for probability weighting are also presented in Fig 1D. The relative amounts of the WX and WY species provide the weighting by catalyzing the AM system to favor X or Y respectively (with the strength of weighting determined by the “bias” rate constant). Specifically, we characterized the switching probabilities of this system as a function of the $\frac{\text{WX}}{\text{WX}+\text{WY}}$ ratio. While some previous work has analyzed the approximate majority system formally in terms of the initial margin of one species required to converge to a consensus decision in that direction [20, 21], here we are interested in obtaining specific *probabilities* of convergence to either species, and we argue that the sizes of the associated state spaces for non-trivial numbers of initial species are far too large to be analyzed in this manner. Therefore, here we take an empirical approach, aggregating probabilities across many simulations of a single probabilistic choice. These results are shown in Fig 2. As expected the probability of choosing X tends to zero as $\frac{\text{WX}}{\text{WX}+\text{WY}}$ tends to zero, and tends to one as $\frac{\text{WX}}{\text{WX}+\text{WY}}$ tends to one. The switching probabilities are non-linear and the shape of the curve is a function of the “bias” reaction rate. This demonstrates that decision probabilities in our system can be straightforwardly tuned by adjusting the quantity of another chemical species in the system; we will exploit this below to enable dynamic training of response probabilities via an operant conditioning protocol.

Furthermore, we characterized the switching probabilities in simulations of multiple sequential decisions. For such simulations that involve multiple sequential decisions, there is the question of how long one must wait following the removal of one stimulus presentation before the next stimulus presentation can begin. To that end, we characterized the required reset time by analyzing the first-passage time of the state species count from 100% of the total to 50%, to demonstrate that sequential decisions are indeed independent, provided that one waits long enough to reactivate the stimulus. From the data presented in Fig A in S1 Text, we conclude that waiting 6 s should suffice to produce independent decisions for *kNoise* = 1. Re-stimulation before the system has reset will create a sort of memory, since the initial state species distribution will be biased toward its previous state, which could be desired for certain applications but that we are not concerned with in this work. Based on this data, we replicated the probabilistic choice experiment from Fig 2 but using 100 simulation traces with 100 decisions per trace, as opposed to 10,000 traces with one decision per trace. Those results are shown in Fig B in S1 Text, along with the same data from Fig 2 for comparison and similar results using 1000 copies of the decision species as opposed to 100. The strong similarity between the statistics for sequential and individual decisions suggests that our system can indeed make multiple sequential decisions in a single simulation run, crucially, with independent probabilities. These results mean that our system could be used over time with dynamically tunable probabilities, thereby enabling our proposed application in adaptive behavioral control of biomolecular systems. We chose to specifically analyze systems containing 100 and 1000 copies of the decision-making molecules because those numbers correspond with the numbers of copies of key molecules present in many simple single-celled organisms, thus demonstrating that our system could be used to implement rationally engineered decision-making in systems at this level of complexity. Adding yet more copies of the molecules would be expected to further smooth out these curves, so we do not simulate such systems directly. Similarly, we focus on analyzing the effect of modulating the *kNoise* parameter on the time to revert back to the noisy indeterminate state because the laws of chemical kinetic imply that simply adding more copies of the X and Y species would just speed up this process yet further.

### Multi-way choice

The systems studied above were characterized for a choice between two options, X and Y. However, the underlying AM system can be generalized to more than two species by simply adding additional reactions to enable the additional species to combine with the others to produce the intermediate B, and also to catalytically recruit that intermediate back to their side. Therefore, we can also straightforwardly generalize our decision-making system to choose between one of three or more possible options, as shown in panel A in Fig C in S1 Text. In this case, we use 99 individuals to simplify the division into three; the equilibrium of the entropy generation module fluctuates around 33:33:33 and whichever of those species is in the majority when the stimulus is activated will come to dominate, producing a 3-way probabilistic system-wide choice, as shown in panel b in Fig C in S1 Text. Furthermore, as shown in panels c and d in Fig C in S1 Text, we can tune the decision-making probabilities in the 3-way choice system using the same weighting approach as above. Thus, we can generalize our system to choose between more than two possible response options with tunable probability. However, we note that the number of distinct possibilities will be limited by the number of molecules in the system, as more more distinct probabilities will require more individual species so that the entropy generation module functions reliably to give reliable probabilities.

### Operant conditioning

**Fig 3:**
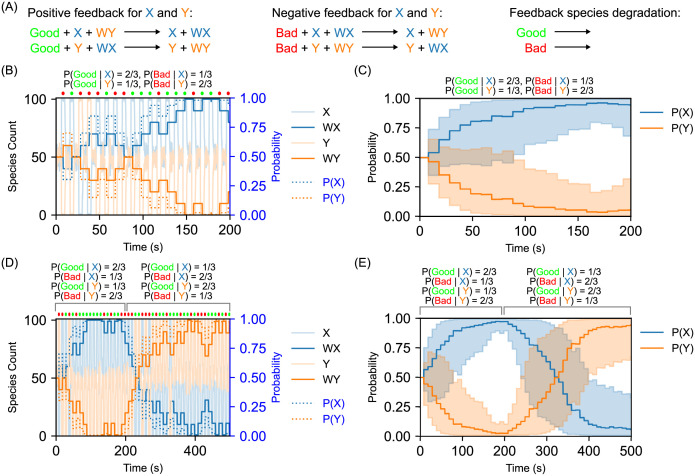
Operant conditioning of stochastic CRNs in environments with probabilistic feedback. (A) Additional reactions to provide feedback response capabilities to the decision-making CRN presented in Fig 1D. The positive feedback species “Good” converts WY to WX in the presence of X and converts WX to WY in the presence of Y. Thus, the WX weight is increased for the next round when “Good” feedback is observed in the presence of the X decision species, and similarly for WY. The negative feedback species “Bad” works in the opposite direction, converting WX to WY in the presence of X (thereby disfavoring the X choice in future rounds) and similarly converting WY to WX in the presence of Y. The feedback species degradation reactions serve to drain away any excess feedback species in the case where there are not sufficient decision or weight species present to drive consumption of the feedback species, thus ensuring that they do not linger in the system and influence future feedback rounds. The rate constants for the positive and negative feedback reactions are *kFB* = 1. The rate constants for the feedback species degradation reactions are *kFBDeg* = 1. (B) Representative time course from an operant conditioning simulation in a constant environment that administers probabilistic feedback based on CRN decisions. Starting with 50 of each weight species, 20 stimulus/relaxation cycles are performed. The environment responds probabilistically by supplying feedback in the form of 10 molecules of either Good or Bad species, based on which of the decision species is above a threshold of 90 molecules at the time feedback is calculated. If decision X was taken, the conditional probabilities of the feedback responses are 2/3 for Good and 1/3 for Bad, and if decision Y was taken, these probabilities are 1/3 for Good and 2/3 for Bad. Rate constant values are as outlined in Fig 1 and above. Here and henceforth, the small discs above the trace show whether positive (green) or negative (red) feedback was administered in that round. Species counts for X, Y, WX, and WY are plotted on the left-hand axis. The corresponding decision probabilities P(X) and P(Y) are plotted on the right-hand axis; these are calculated by using the “*kBias* = 30” transfer curve from Fig 2 as a calibration to convert weight species counts into the corresponding probabilities. In this example, positive and negative reinforcement from the environment drives the system to favor the X response as this is more likely to elicit positive feedback and less likely to elicit negative feedback. (C) Results from 50 distinct stochastic simulations of the conditioning experiment from part (b), plotting the averaged decision probabilities only. Line indicates the mean; shaded area represents one standard deviation above and below the mean. (D) Representative time course from an operant conditioning simulation in a dynamic environment that administers probabilistic, time-dependent feedback based on CRN decisions. Starting with 50 of each weight species, 50 stimulus/relaxation cycles are performed. Initially, the environment responds with probabilities as in part (b), however, at time *t* = 200 the probabilities are switched. Rate constant values are as outlined in Fig 1 and above and probabilities are calculated as described in part (b). In this example, the change in the response probabilities at time *t* = 200 demonstrates the ability of the system to adapt to this change and switch itself from a high probability of the X response to a high probability of the Y response. (E) Results from 50 distinct stochastic simulations of the conditioning experiment from part (d), plotting the averaged decision probabilities only. Line indicates the mean; shaded area represents one standard deviation above and below the mean.

The system thus far is capable of executing a probabilistic choice, whose probability is a function of the number of weight species corresponding to each choice. We envision this system existing in an environment where its choices result in some chemical feedback from the environment. This is a simplified system that we use here to model more generalized and indirect notions of environmental feedback, that we envision could result in similar conditioning of a synthetic cell system. For example, the environmental feedback could take the form of the synthetic cell taking an action which changes the chemical environment in a manner that is sensed via chemical receptors in the synthetic cell and hence transduced into an interpreted positive or negative feedback. Here, we implement operant conditioning by introducing reactions that use this feedback to update the weight species. By conserving the number of weight species, we ensure the probability of the system’s choices are predictable according to the transfer curve shown in Fig 2. Our simple operant conditioning feedback reactions act on only two types of feedback species, Good and Bad. The Good and Bad species will be consumed by competing reactions catalyzed by the state species, consuming a weight species and producing another weight species of a different type. It is important to note that the feedback reactions are identical regardless of the feedback regime used by the external environment; therefore, any changes to the responses of the system are not hard-coded within its network structure but are rather determined via interactions with the environment, which thus indirectly controls its behavior via how the decision making system is conditioned. The reactions used to process feedback are shown in Fig 3A. These are trimolecular reactions in which the Good and Bad species act in concert with a state and weight pair. The reactions are agnostic to the current state of the system and to the environment, and will work as intended given the correct context. The Good reactions fire when a Good molecule encounters any state species and any non-identical weight species, converting the weight species to match. The Bad reactions act on a matching state and weight pair, converting the weight species to a non-matching weight. There exist reactions for all combinations of weights and states, so the system has no inherent bias as to where weights are reallocated when Bad feedback is detected. The environment may attempt to give a response at any time by inspecting the state molecules; however, if feedback were introduced during a period without Stimulus, the weight species would be reallocated unpredictably. We define the environment to give a response only if a given state species is above a threshold that we define as 90 molecules or more in a system with 100 state molecules, which will therefore correspond to a consistent and stable state representation. By enforcing this threshold, we ensure that when the environment provides feedback, the Good reactions are likely to reinforce the weight associated with the current state, and Bad reactions are likely to distribute weight away from the current state. We define the environment as the set of responses for each state. This definition of the environment is simple so as to provide an overall system that is relatively simple to implement and analyze. We note that real-world environments would not be so clean in their responses as the systems imagined here. For example, the threshold on feedback outlined above could model a more realistic sigmoidal response curve that may be factored through signal processing steps to sharpen the on/off transition before being passed along to a decision-making system such as that defined here. In addition, while our examples here show all stimulus and feedback timings following a regular, periodic schedule, in actual fact this is not required, provided that the feedback signal arrives while the stimulus is still present so that the system can process them as a pair. We return to such design issues in the Discussion below. In our initial work on this system, we explored simple environments that provide deterministic feedback on system responses, as outlined in Fig D in S1 Text. In these and subsequent simulation, the environment attempts to respond at regular intervals at the midpoint of each window of time during which Stimulus is present. The results from this preliminary work indicated that the weights in our system can be conditioned in a particular direction based on feedback from a deterministic environment.

### Adapting to complex environments

Real environments, however, are neither simple nor deterministic. Therefore, we next studied our system in environments that provide time-dependent and probabilistic feedback. In Fig 3B and 3C, the environment sometimes reacts to state X with Good feedback (with probability $\frac{2}{3}$) and sometimes with Bad feedback (with probability $\frac{1}{3}$) and reacts to state Y with inverse probabilities. That is to say, the environment generally favors state X and as expected, the system is conditioned to increase the weight WX after multiple choices. Essentially, the weights perform a random walk, taking a step each time feedback is provided by the environment. The probability of taking a step in a given direction is conditional upon the current state, where the prior probability of being in a given state is a function of the current weights, and the environment encodes the conditional probabilities of the random walk. If the environment favors a certain state as Good and another as Bad, then the weights will converge to that state. This is confirmed both by Fig 3B, which presents a single representative stochastic simulation trace of this system, and by Fig 3C, which presents statistics showing how the decision probabilities change over time when averages over 50 stochastic simulations.

Building on those results, we next tested the behavior of our system in dynamic environments where the feedback probabilities change over time. In principle, our operant conditioning system should be able to change its behavior over time in response to changing environmental feedback. Indeed, our results from Fig 3D and 3E show that if the environment initially favors one state and then later favors the other state then the system will autonomously adapt its behavior to obtain positive feedback as the feedback probabilities change over time. As above, Fig 3D shows a single representative simulation trace while Fig 3E presents statistics averaged over a battery of simulations. No additional chemical reactions are needed to produce this behavior; it arises naturally from the properties of the operant conditioning system that we have developed.

The behavior of the system is almost entirely determined by the feedback strategy of the environment. The tunability in the CRN comes from the ratio of the rates of feedback being consumed versus degraded. This can be adjusted to suppress the amount of feedback consumed, but can not result in amplification of feedback if there is a small amount of feedback given. Therefore, the maximum number of changed weights is equal to the number of feedback molecules given. Note that in Fig 3E, it takes a certain amount of time for the weights to reflect the new environmental feedback strategy. Based on the amount of feedback and the frequency with which the environment changes strategies, the behavior of the system may not reflect the new environmental feedback if it only exists transiently and with a small amount of feedback; in a sense this should provide a “temporal band-pass filter” that ignores such transient changes in the environment. We can observe this effect to some extent in Fig 3E, where the decision probabilities switch at time *t* = 200 but it takes almost 150 additional time units for the decision probabilities to switch past 50:50, on average. If the feedback probabilities were switched back during that time, one would likely observe relatively few Y decisions before reverting to the state where X dominates.

### Response generation

**Fig 4:**
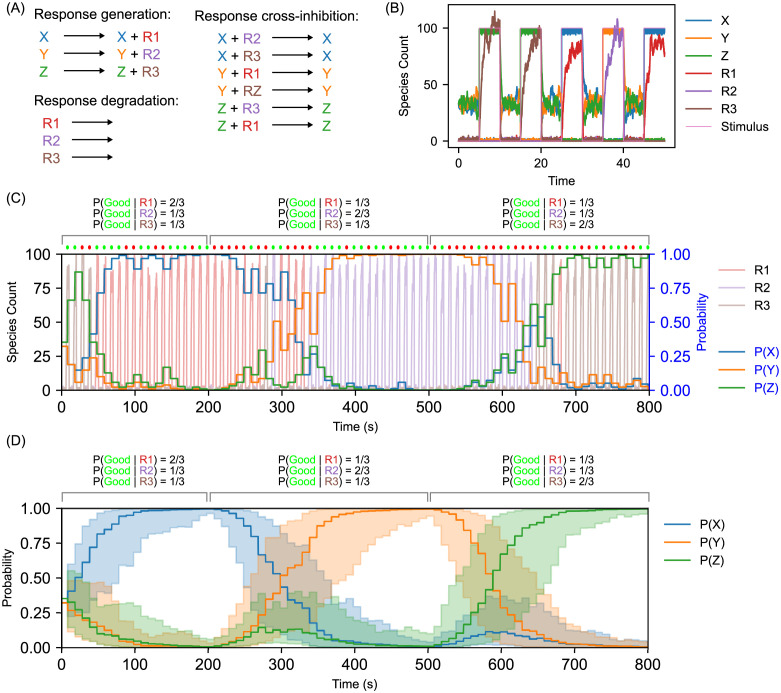
Generation of distinct response species from a ternary probabilistic decision-making CRN. (A) Additional reactions that can be added to the ternary decision-making CRN that link the decision species X, Y, and Z to the production of distinct “response” species R1, R2, and R3, respectively. Via the response generation reactions, each of the decision species catalyzes generation of the corresponding response species. Via the response cross-inhibition reactions, each of the decision species catalyzes degradation of the “other” response species. Finally, the response species also degrade via the response degradation reactions. The rate constants for the response generation reactions are *kResponse* = 1. The rate constants for the response cross-inhibition reactions are *kResponseRepression* = 1. The rate constants for the response degradation reactions are *kResponseDecay* = 1. (B) Representative time course illustrating the link from decision-making to the generation of distinct response species. When the decision-making species are at roughly equal counts during the entropy generation phases, the response species are generated but then immediately degrade each other, and thus the overall levels of the response species remain low. However, when a decision is taken in response to a stimulus and one decision species comes to dominate, it is able to promote generation of the corresponding response species while suppressing generation of the others. Thus, we can implement response generation that matches the decision but is expressed via a distinct abstract chemical species, which could represent an actuator. Initial species counts in this simulation were WX=WY=33 and WZ=34. (C) Representative time course from an operant conditioning simulation in a dynamic environment that administers probabilistic, time-dependent feedback based on observing the generation of response species by the CRN. Starting with decision species counts of X=Y=Z=33 and initial weight species counts of WX=WY=33 and WZ=34, 80 training cycles were run, with a stimulus of 100 molecules on for 4 time units and then off for 6 time units per cycle. The environmental feedback was a probabilistic, time-dependent response, similar to that from Fig 3 except that the feedback is now based on observed the response molecules, R1, R2, and R3, instead of the decision molecules directly. The threshold for determining which feedback to supply was set at 40 of one of the response molecules present; this is lower than the earlier simulations due to the greater stochasticity in the steady-state levels of the response species. When 0 < *t* < 200, the environment responds to R1 with 10 Good molecules with probability 2/3 or with 10 Bad molecules with probability 1/3. In the same interval, the environment responds to R2 or R3 with 10 Good molecules with probability 1/3 and 10 Bad molecules with probability 2/3. These probabilities cycle to R2 being favored when 200 < *t* < 500 and to R3 being favored when *t* ≥ 500. (We only report P(Good|Rx) in the figure; P(Bad|Rx) can be calculated straightforwardly as 1-P(Good|Rx) for each response species Rx.) This simulation demonstrates the favored response shifting from R1 to R2 to R3 as the feedback from the environment changes over time. (D) Results from 50 distinct stochastic simulations of the conditioning experiment from part (d), plotting the averaged decision probabilities only. Line indicates the mean; shaded area represents one standard deviation above and below the mean.

We wanted to show that our system can be used not just to pick between the species used in the AM reaction but also to use the decision to drive external behaviors via the generation of arbitrary “output” or “response” species, by linking each AM species (X, Y, or Z, say) to a corresponding “response” species (R1, R2, or R3). This can be achieved via additional chemical reactions outlined in Fig 4A, in which X catalyzes production of R1 but degradation of R2 and R3, and similarly for Y and Z. These response generation and response degradation reactions ensure that in the entropy generation phase where all AM species are present in roughly equal amounts, the generation of all response species is inhibited and their numbers remain low. When one of the AM species is selected in response to the stimulus, however, it is able to catalyze promotion of the corresponding response species while keeping the others repressed, and that response species goes high. When the stimulus is removed, the response species will decay as the other AM species come back up to fluctuate around the equilibrium. The simulation shown in Fig 4B illustrates this approach, which demonstrates that our probabilistic decision-making CRNs can be linked to arbitrary external processes via the generation of distinct response species upon stimulation.

In a real-life system, environmental feedback would be provided on the basis of such observable responses from the system, rather than on the internal decision variables used within the CRN. We therefore sought to demonstrate a response-based feedback system similar to that shown in Fig 3C and 3D. Representative simulation traces and population averages of the decision probabilities are presented in Fig 4C and 4D, respectively. Here, the environment is now responding to the response species instead of the state species. As the response species are a deterministic function of each state species, the environment is able to condition the system as though it were responding directly to the state species. This changes the calculations done to compute the environmental response but does not require any changes to the feedback processing reactions within the CRN itself. As this example involves a three-way choice, we are able to condition responses R1, R2, and R3 in order. This work demonstrates that our system can be conditioned indirectly via a chemical species not involved in the decision process itself. In theory, the environment could respond to any species downstream from the state species, so long as its feedback is given as Good and Bad species while the original upstream stimulus is still present.

### Weight resetting module

In all previous examples with feedback, the system always tends toward maximal bias of one state. This is because the environment provides positive reinforcement of at least one state, and the maximum amount of weight species ensures nearly 100% bias according to the transfer curve shown in Fig 2. There may be some situation where it is desired that the system does not converge to a 100% bias. This could be achieved by choosing a flatter transfer curve, however, it is also of interest to consider other mechanisms by which the state of the system could be reset independently of the environment.

**Fig 5:**
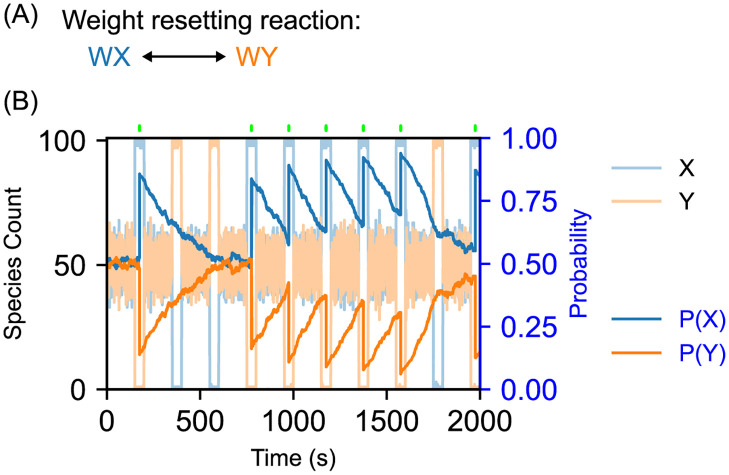
Forgetting weights in operant conditioning systems. (A) Additional reactions to incorporate forgetting into a simple binary probabilistic choice system. The rate constants for the forward and backward reactions in the weight resetting reactions are *kWeightReset* = 0.003. By enabling the weight species to interconvert at a low rate, they will tend to revert to the evenly-distributed equilibrium when conditioning reactions are not driving them away from this state. Thus, if no relevant feedback is provided over some time period, the system will tend to “forget” the trained weights. (B) Representative time course from an operant conditioning simulation with weight forgetting. This uses a simple binary choice system augmented with the weight resetting reactions outlined in part (a). Starting with 500 copies of each weight species, 10 stimulus/relaxation cycles are performed with 50 time units of Stimulus and 150 seconds of relaxation. The environment responds to state X with 200 Good molecules and does not respond to state Y at all. Rate constant parameters used in this simulation were *kBias* = 3, *kAM* = 1, *kNoise* = 1, *kFB* = 1, *kFBDeg* = 1, *kWeightReset* = .003. Positive feedback on the initial choice of X causes P(X) to increase, but the lack of feedback on subsequent choices of Y causes P(X) to decay back to the equilibrium state; a subsequent run of X choices causes P(X) to increase and remain high while positive feedback is continually received. (For this simulation, the appropriate transfer curve from Fig B from S1 Text for 1000 total weight molecules was used to calculate decision probabilities from the weight species counts. A total count of 1000 weight species was used so that a higher weight-resetting rate could be used, ensuring a more consistent decay curve.)

As the state species participate in the entropy generation reactions shown in Fig 1E, they tend toward a 1:1 ratio. Similarly, we can introduce resetting reactions for the weight species so that the bias tends toward 50% for each state in the absence of feedback perturbations from the environment. Fig 5 shows an example trace for this type of system, where a large amount of feedback is given only when X is encountered by the environment. There are three main parameters that affect the behavior of the system given this additional module: time between perturbations, rate constant of the weight resetting reaction, and amount of feedback given by the environment. As the time between perturbations grows relative to the time needed for the weights to reach equilibrium, each choice becomes less dependent on the previous. By decreasing the time between perturbations or increasing the amount of response given by the environment, a given choice becomes increasingly dependent on its previous choice. In Fig 5 we chose to tune the parameters such that a single choice of X tends to result in a cluster of repeated X choices, but returns to an unbiased system whenever Y is chosen once.

## Discussion

In summary, in this work we have presented the design and analysis of a set of simple yet powerful molecular programming primitives for probabilistic choice and environmental adaptation in chemical reaction network systems. We have shown that population-level decision-making can be weighted and that these weights can be conditioned via feedback from an external environment, leading to non-trivial yet autonomous behaviors, such as the ability to adapt to dynamic environments where feedback changes over time.

### Exploring the design space for decision-making CRNs

We have outlined a toolkit of modules that enable probabilistic consensus of a population with dynamic control over the weighted probabilities. However, there are many functionally identical or similar networks that could replace the various modules in our architecture. These other modules may add layers of tunability or new functionality, but can also introduce additional complexity and noise. Here we will discuss some of the possible design space related to various modules.

The most important module in our system is the approximate majority module. This enables consensus and can be made prone to error in selecting the initial majority, which we exploit as a feature enabling tunable probabilistic choice. In our version, the number of state species is conserved. This is not realistic in many biological contexts including nucleic acid based computing systems because of enzymatic degradation. To model this, we could add another module with degradation reactions for the state species and a replenishment reaction for the intermediate B species. Then, there would be some non-conserved quantity of state species that fluctuates about some steady state as a function of the ratio of replenishment to degradation. The fluctuations would introduce noise that would affect the relative strength of the weight reactions. The system would be less precise, but the steady state would now be a tunable parameter as a function of the rate constants in the degradation and replenishment reactions. Then we could add catalysts to tune the effective rate of replenishment and degradation, and the catalysts could be conditioned via environmental feedback in the same way as the weight species.

The entropy generation module is a single reversible reaction that inter-converts the state species, which results in a steady-state 1:1 ratio of state species in the absence of stimulus. Another possibility, which is outlined and simulated in Fig E from S1 Text, is to convert all state species to intermediate species at some constant rate, so that the steady state in the absence of stimulus is a population consensus of the intermediate species B. We call this the “decision erasure” system, as these reactions cause the decision (X or Y) to be erased when these species are all converted back to B. This requires the bias reactions to also be catalyzed by stimulus, otherwise the state species would be converted by the bias species in the absence of stimulus. The benefits of this system are that there are no state species present in the absence of stimulus, which may be of interest in a system where continual presence of state species is not desired. Another benefit is that when stimulus is introduced, the system begins from the same state every time instead of starting with a random amount of each state species, resulting in more predictable decisions as a function of the number of weight species. The downside of this system is that instead of a single reversible reaction between X and Y, there are multiple irreversible reactions where each reactant is driven to the same product, and would therefore require some supply of consumable fuels in a DNA implementation.

Another element to consider is how to redistribute the probability mass in the face of negative feedback from the environment. The negative feedback reactions in Fig 3A distribute probability uniformly. In that example, the negative feedback reaction shown consumes a molecule of WX and converts it to WY. However, if there were three states, X, Y, and Z, there would be a competing reaction that converts a molecule of WX to WZ, so the negative feedback module would distribute probability to the other possible states uniformly. This corresponds to a prior in which no assumptions are made about the correct state when negative feedback is provided. However, the reactions in this module could be modified to distribute probability as a function of the state species consumed and could be used to prioritize which state the weights are allocated toward when negative feedback is consumed.

Finally, the response generation module shown in Fig 4 uses competing rates of generation and degradation to define a steady state of response species and fluctuates around 100 molecules in this example. We could introduce a catalyzed version of the degradation reaction, so that the steady state becomes a parameter tunable by the amount of catalyst present. This would allow a response level that can participate in a feedback control system. We have only shown very simple environmental feedback strategies; a more complex strategy might produce feedback proportional to the amount of response observed. In summary, there are a wide range of possible variations on the scheme presented here that could be explored in future work.

### Related work in molecular programming

Fett et al. [24] proposed a system similar to ours that starts with a heterogeneous population and produces a population of a single molecular species. The output species is chosen probabilistically as a function of the ratio of molecules in an initial population of upstream reactants. That system exploits the fact that the first reaction to fire in a CRN is distributed according to the relative rate constants and reactant counts. In this initial reaction set, each reaction has its own corresponding output catalyst and downstream output. Once the first reaction fires, the output is quickly amplified and the competing reactions are suppressed. This is similar to how our system chooses the consensus as a function of the initial population of weight and state molecules, and how the response reactions cross-inhibit each other; however, in their system, the final population being amplified and downstream from the reactions that encode the probabilities means that the system can not reset and perform another choice. We instead abstract the probabilities into their own module that biases the outcome of the approximate majority CRN, which probabilistically amplifies the population in the majority while conserving the molecular count of its reactants and products, allowing the system to be reset easily with entropy generating reactions.

Wilhelm et al. [14] constructed an experimental DNA strand displacement circuit system implementing probabilistic switching, in which a single input molecule will produce any one of multiple outputs with tunable probabilities. While the output for a given input molecule is probabilistic, this produces a deterministic heterogeneous population, in which the population of each output is proportional to the probability of being produced for a single input molecule. In contrast, our system proposed here produces probabilistic switching of the entire population into one output state or another. Related work by Rodriguez et al. [25] provides an example of an experimental chemical system where the initial minority wins out by design, which is another important aspect of our work, although that system is not probabilistic in nature.

Previous work on learning in chemical systems has focused on a variety of tasks and learning algorithms. We, and others, have implemented a number of supervised learning algorithms as CRN designs [26–28]. However, supervised learning algorithms lack the simplicity and flexibility of conditioning-based approaches such as that proposed here. Banda et al. [29] used reinforcement-based techniques to teach a simulated CRN the weights to implement Boolean functions including XOR. That work is similar in some respects to the work reported here, except that our system conditions the weights in a probabilistic decision-making system rather than in a deterministic function approximation module. Thus, our work is arguably more applicable to modeling the decision-making behavior of autonomous biochemical agents. Poole et al. [30] demonstrated CRN implementations of Boltzmann machines, a stochastic neural network capable of inference of probability distributions; subsequent work showed that CRNs can be programmed to generate discrete probability distributions [31], including with guarantees of robustness to perturbations [32]. Furthermore, intriguing applications of stochastic chemical reaction networks to search for solutions to a range of problems, including boolean satisfiability, were also recently reported [33]. While it is known that stochastic systems can exhibit behaviors that are “deviant” with regard to those predicted by deterministic chemical kinetics [34], this work hints at fascinating connections between stochastic CRNs and learning and adaptation that may allow the unique capabilities of molecular-scale information processing to be harnessed in a natural manner. Another prime example of this is recent work on chemical implementations of spiking neurons [35] which can learn a variety of correlations between signals based solely on temporal correspondence. This lends further credence to the notion that such approaches to semi-autonomous molecular learning and adaptation, similar to our approach described in this paper, may be a natural fit for the hardware available to molecular programmers.

Finally, in previous work [36] we reported stochastic CRNs that implement deterministic state transitions between population states that are stabilized by the approximate majority CRN. The AM reactions are not catalyzed in that system, so the system is never resting in a non-consensus state; in other words, the state is persistent. A spike of input species will interact with a given state species to induce a transition to the next state encoded as defined by the finite state automata. The system outlined here could be used in conjunction with that work to create finite automata where the next state is chosen probabilistically.

### Relevance to systems and synthetic biology

As outlined above, we primarily envision that our AM-based system can be viewed as a potential control system for synthetic cells. The system proposed here is at the highest level of abstraction as a chemical reaction network with abstract chemical species. Having identified a relatively minimal set of interactions from which tunable probabilistic choice can be implemented, there are components of natural systems that implement the same behavior as the individual modules in our system. Therefore, it may be possible to harness existing modules to complete the behavior of our proposed system. For example, Dodd et al. [37] study a simplified model of nucleosome modification in which a nucleosome can be either in an unmodified state or in one of two modified states. The modified states in their model are analogous to what we call *state* species and the unmodified state is like our *intermediate* species. The rates of recruitment and noise can be adjusted to achieve a bistable system where all nucleosomes form a consensus of a single modified state. This system therefore implements the core module of our system, the approximate majority, and it might be possible to build around this to complete the tunable probabilistic choice mechanism. Furthermore, the AM system used in our simulations is just one possible architecture for implementing probabilistic chemical decision-making, but there exist alternative AM systems using trimolecular reactions [21] as well as more complex biological switches that have been identified in natural systems and studied in terms of cellular computation [23, 38]. These could also be used in an operant conditioning system similar to ours. We note also that rationally designed enzyme-based strategies have also recently been used to implement abstract CRNs [39, 40], which indicates another possible future direction for the implementation of adaptive decision-making systems such as ours in a *synthetic* biology context.

We also note that motifs implementing the underlying approximate majority algorithm have been found in biological networks including the cell cycle switch [31]. We refer the reader to Cardelli et al. [23] for a review of the links between biological switches and population protocols from computer science, in particular, the AM switch. This hints that biology may already have developed probabilistic decision-making systems such as that proposed here, perhaps even with corresponding feedback loops to permit adaptation; this would be a fascinating topic of future study in *systems* biology. In particular, this work also highlights the possibility of implementing the abstract CRNs proposed here biochemical implementation strategies closer to those used to implement information processing in living cells, such as genetic regulatory networks [41] and/or protein phosphorylation networks [42]. In this conception, one might imagine a genetic implementation of the bistable switch, such as those proposed by Cardelli et al. [23], with fast phosphorylation and dephosphorylation reactions perhaps providing the on/off switch for this decision-making system in the presence or absence of the triggering stimulus and/or providing the entropy generation that enables a random decision to be made in the first place. There is an extensive literature on decision-making in systems biology: network motifs [41] are small subgraphs of a genetic regulatory networks that are enriched in naturally occurring networks relative to the number of occurrences that would be expected in a randomly assembled graph of similar size; their enrichment suggests that the motif serves some useful information processing function for the host cell. Early work in this direction outlined a number of genetic circuit motifs capable of memory after stimuli are removed, such as positive feedback gene expression cycles [41] and bistable switches, which constituted some of the earliest work in the field of synthetic biology [43]. Biologists have also long studied other decision-making switches such as ultrasensitive MAPK cascades [44]. Finally, other related work in systems biology has explored systems capable of bistability, such as the Schlögl model [45], which is an autocatalytic system consisting of the following reactions:

$$A+2X\overset{k_{1}}{\underset{k_{2}}{⇌}}3X\;\;\;\;\;\;B\overset{k_{3}}{\underset{k_{4}}{⇌}}X.$$

This system uses trimolecular reactions, as does ours, though it is worth noting that in our trimolecular reactions that third reactant is a catalyst that is distinct from the other species involved in the reaction. (Furthermore, such a catalyst could be implemented in an experimental system as a non-chemical input, perhaps as an optical signal that enables the corresponding reactions to proceed.) More recent theoretical analysis of this system [46] has studied it in the deterministic as well as the stochastic regime and derived analytical expressions for the time spent in each of the bistable states. Thus, this system could provide an alternative basis for a probabilistic decision-making system in which there is a known analytic expression to derive the expected probability of being in a particular state when the stimulus is switched on. This highlights the broad range of potential designs for decision-making modules that could be incorporated into a system capable of being conditioned in the manner we explore here.

### Future directions

The system design explored here represents the simplest conception of operant conditioning, in which the environment provides feedback on the last stimulus-response pair only and the system itself has no memory of prior cycles. As our CRN design is completely agnostic to the behavior of the environment, and given the flexibility available to us in the specification of environmental responses, in principle the environment could remember past cycles and adjust its feedback based on that memory, which would not even require any changes to be made to the CRN itself and could lead to more nuanced conditioning of circuit responses. Alternatively, or in addition, the CRN component itself could be modified to remember past environmental feedback, perhaps using a chemical implementation of a delay line [47]. Then, the feedback signals provided by the environment could be modulated internally by the chemical system as appropriate to produce more sophisticated behavioral changes. This example highlights the somewhat fuzzy line dividing the computation being carried out in the chemical system versus that being carried out with a nebulous external environment. In this work, we choose to consider the environment as a black box that encapsulates everything involved in the adaptive process that is not the core decision-making circuitry of the system, and thus can be thought of as comprising various aspects of a synthetic cell itself (including receptors and possibly some signal pre-processing networks) as well as external factors from the environment that produce an effect on the system based on the system’s stimulus-response behavior (such as the effect of a motile synthetic cell moving to a new location where the chemical environment differs, for example). An important direction of future research would be to design and implement systems that directly model as much of the sensing, information processing, and response aspects of the synthetic cell as possible, and minimize that which is covered by the black box of the “environment”. Furthermore, for any practical deployment a system such as ours would need to be robust to less perfect environments, which may not adhere to a strict response-feedback regime such that assumed here, and where the stimulus signal may be a less perfect digital waveform than those shown here. Such imperfections in the stimulus and feedback signals could be accommodated within the CRN component of the system by building thresholds into the information processing capabilities of the decision-making CRN itself; previous work in the context of DNA strand displacement have shown how such thresholds can be implemented by exploiting the separation of timescales between different classes of reactions [9].

The abstract chemical reaction proposed herein contains relatively few reactions and could, at least in in principle, be translated into a wet chemistry implementation using DNA strand displacement reactions [13]. Indeed, a single-use implementation of the AM module has already been demonstrated using DNA [10] and recent advances in DNA circuit implementation mean that increasingly large reaction networks are becoming feasible [12]. In terms of a practical application, our system could perhaps be used to control the behavior of a synthetic or rationally engineered unicellular organism, for example mimicking avoidance behaviors observed in *Stentor* cells [3]. The cell first assumes one state (bending) in response to a noxious stimulus in an attempt to avoid it. Then if the stimulus continues, the cell will assume a second state (reversal of ciliary action), and again a third (detachment from substrate) until the stimulus stops. In *Stentor*, repeated stimulation by the noxious stimulus results in the cell being more likely to skip the first avoidance state and move directly to the more extreme measures of the second or third state, via a weighted probabilistic decision-making computation. In our model, the repeated noxious stimulus would increasingly modify the bias for transitioning to the second and third states, while perhaps forgetting this conditioning after some time as shown in Fig 5. The networks proposed here thus provide a relatively simple decision-making system that could be used to control engineered synthetic cells that include DNA components [48], and may by this mechanism provide some insight into the adaptive behaviors of biological cells, and might also be used to control rationally designed and engineered biorobots made of cellular components such as xenobots [49, 50].

### Materials and methods

All simulations were carried out using ProBioSim, a custom-made Python stochastic simulator (https://github.com/matthewlakin/ProBioSim) that implements Gillespie’s algorithm [51]. This simulator allows us to define not only the chemical reactions and their rates but also perturbations that are calculated as runtime using arbitrary code passed into the simulator as Python functions. This allows the environment to introduce an arbitrary amount of any species at an arbitrary time during the simulation based on the current simulation state at that time, thereby modeling environmental feedback based on probabilistic decisions taken by the chemical reaction network component.

For each set of simulations, a set of seeds are saved to initialize the random number generator (using the random modules from the Python standard library) for each trace, so all data can be reproduced. Simulations may be sampled at a set rate, or they may record every reaction that takes place. In generating the transfer curves, traces were sampled at 100 samples per unit time, except for the traces with 100 cycles each, which were sampled at 10 samples per unit time. The transfer curves have data points at 25 evenly spaced locations on the x-axis, and are connected by straight lines to approximate the continuous curve.

## Supporting information

S1 Text Additional data plots. (PDF)
