# Supplementary material for: Operant conditioning of stochastic chemical reaction networks
Source: PLoS Comput Biol. 2022 Nov 18;18(11):e1010676. doi: 10.1371/journal.pcbi.1010676 (PMC9718418; doi:10.1371/journal.pcbi.1010676)
Supplement: S1 Text — (PDF) [file pcbi.1010676.s001.pdf]

Operant conditioning of stochastic chemical reaction networks
SUPPORTING INFORMATION

David Arredondo[1] and Matthew R. Lakin[1,2,3]

[1]Center for Biomedical Engineering, University of New Mexico, Albuquerque, NM 87131, USA
[2]Department of Computer Science, University of New Mexico, Albuquerque, NM 87131, USA
[2]Department of Chemical & Biological Engineering, University of New Mexico, Albuquerque, NM 87131, USA

October 28, 2022

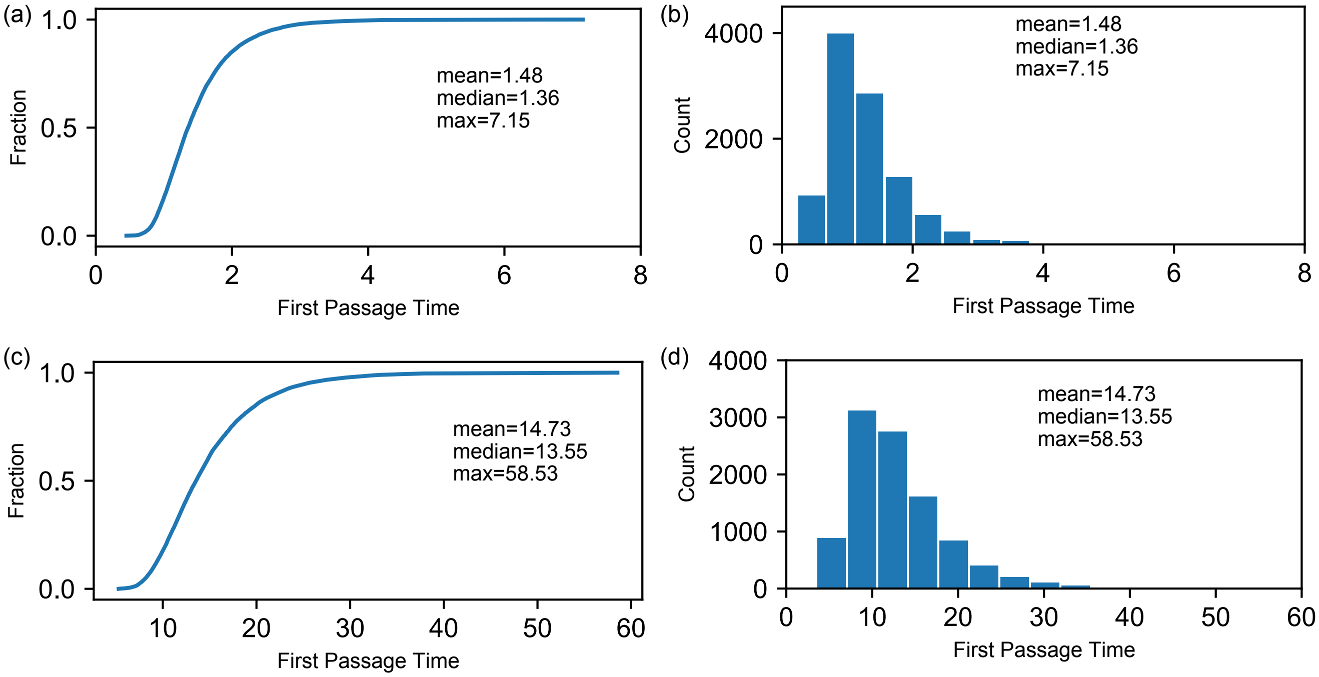

Fig A: Results of first passage time analyses for determining appropriate relaxation times for sequential decision-making simulation experiments. (a) Cumulative distribution function of first passage time to 50 molecules each of X and Y, starting from 100 copies of X, with rate constant $kNoise = 1$. (b) Corresponding histogram of first passage time to 50 molecules each of X and Y, starting from 100 copies of X, with rate $kNoise = 1$. (c) Cumulative distribution function of first passage time to 50 molecules each of X and Y, starting from 100 copies of X, with $kNoise = 0.1$. (d) Corresponding histogram of first passage time to 50 molecules each of X and Y, starting from 100 copies of X, with $kNoise = 0.1$. These results illustrate the length of time required to ensure a reliable return to the equilibrium state, thereby ensuring that subsequent decisions are taken independently.

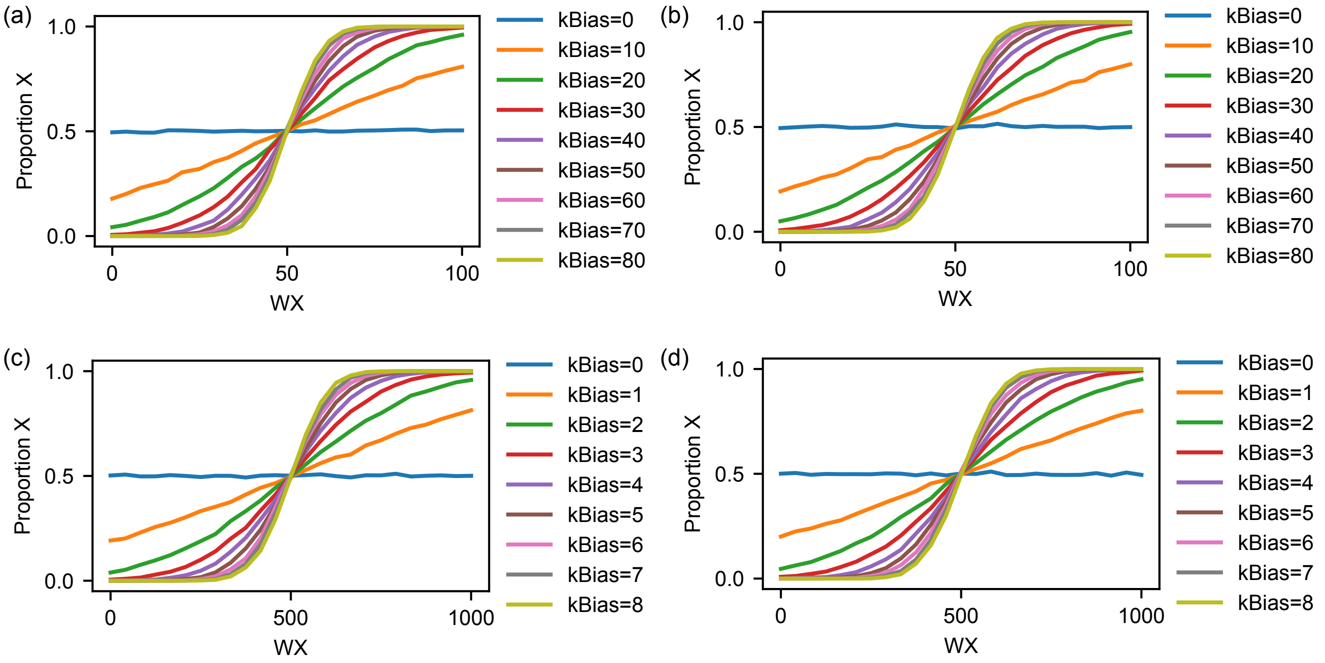

Fig B: Additional analysis of decision probabilities as a function of weight species counts, for various numbers of weight species. Also compared are individual decisions versus multiple sequential decisions; the fact that these plots are so similar demonstrates that the sequential decisions taken in such simulations are independent. (a) In each of 10^5 traces, a CRN is initialized with 50 of each state species X and Y, and a various amount of weight species WX (x-axis) and WY, where $\mathrm{WX} + \mathrm{WY} = 100$. Each trace is 0.5 time units. After 0.45 time units, 100 molecules of the Stimulus species are added. The chosen state is recorded, where a state is defined by 90 or more state molecules when sampled at $t = 0.475$ (halfway between the Stimulus perturbation and the end of the simulation). The proportion of traces that resulted in the X state is shown on the y-axis. The rate *kBias* is varied, and the common parameters are as follows: $kAM = 1$, $kNoise = 1$. *(Note that this figure is identical to Figure 2 from the main text, and is included here for ease of comparison with the other components of this Figure.)* (b) A similar analysis for 100 decision-making cycles in each of 100 traces and where $\mathrm{WX} + \mathrm{WY} = 100$. The rate *kBias* is varied, and the common parameters are as follows: $kAM = 1$, $kNoise = 1$, $kStimulus = 0.1$. The fact that this plot is similar to the plot from part (a) implies that, regardless of whether we take single decisions independently in separate traces or with a large number of decisions in a single simulation, the decisions probabilities are the same. (c) A similar analysis with one decision-making cycle in each of 10^5 traces and where $\mathrm{WX} + \mathrm{WY} = 1000$ (a higher total amount of the weight species). The rate *kBias* is varied, and the common parameters are as follows: $kAM = 1$, $kNoise = 1$, $kStimulus = 0.1$. (d) A similar analysis for 100 decision-making cycles in each of 100 traces and where $\mathrm{WX} + \mathrm{WY} = 1000$. The rate *kBias* is varied, and the common parameters are as follows: $kAM = 1$, $kNoise = 1$, $kStimulus = 0.1$. Again, the similarity to the trace from part (c) implies that sequential decisions are taken independently.

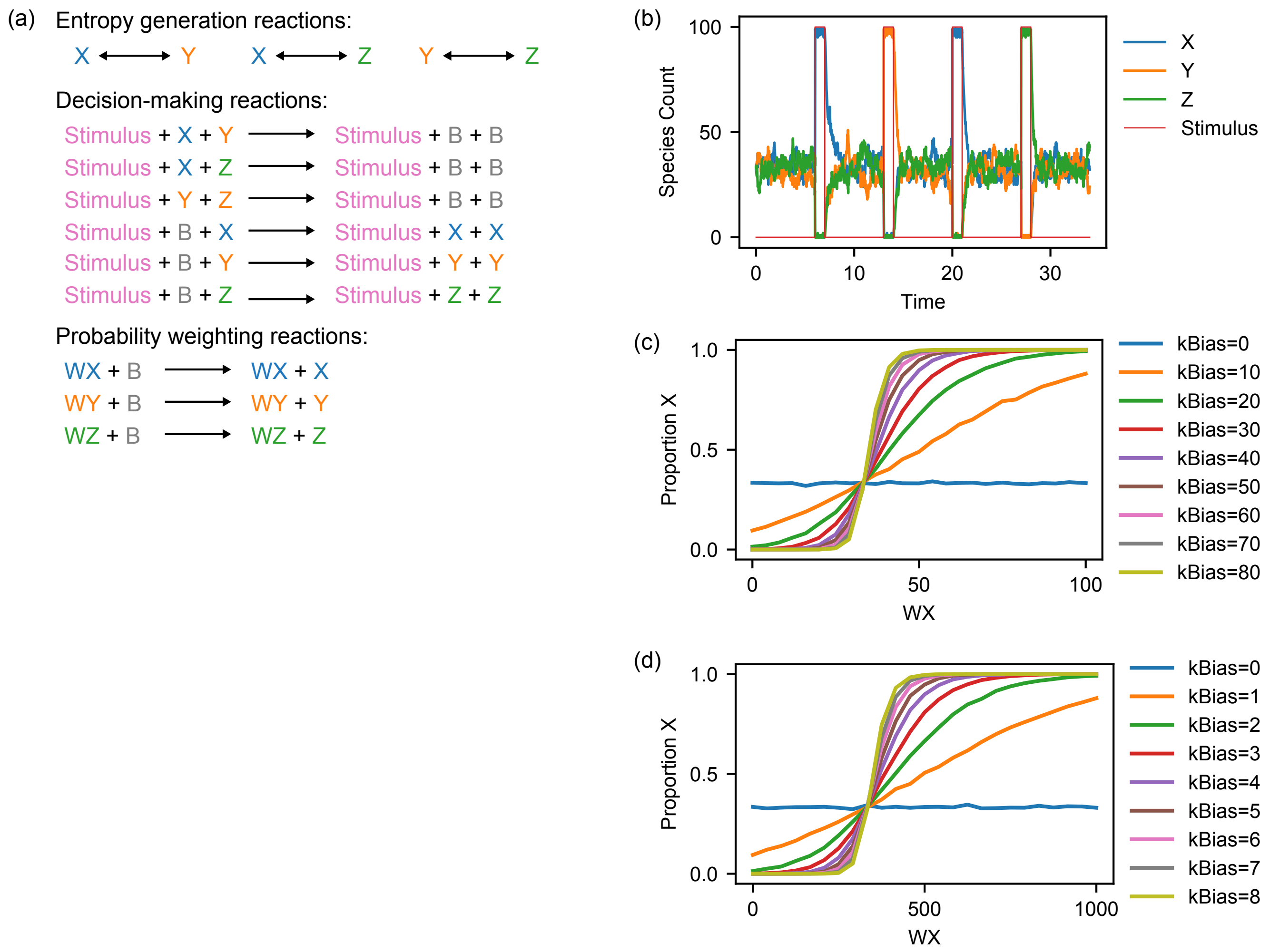

Fig C: Generalization to a ternary probabilistic decision-making system. (a) Abstract chemical reactions specifying the three-way decision-making system. Additional reactions are added to enable interconversion of all three state species, (X, Y, and Z). The "standard" AM system is also augmented to add a third output option, Z. The forward and backward rate constants for the entropy generation reactions are both *kNoise*, the rate constants for the decision-making reactions are *kAM*, and the rate constants for the probability weighting reactions are *kBias*. (b) Example trace illustrating several probabilistic decisions taken by this system with equal probabilities of all three outcomes. This simulation uses rate constants $kAM = 1$, $kNoise = 1$, and $kStimulus = 0.1$. (c) Transfer curve illustrating the probabilities of choosing one outcome (X) in a ternary system as the amount the corresponding weight species (WX) increases. The remaining weight species are divided equally between the two other options, i.e., $\text{WY} = \text{WZ} = (100 - \text{WX})/2$. This simulation uses rate constants $kAM = 1$, $kNoise = 1$, and $kStimulus = 0.1$, and the probabilities are calculated with one decision cycle per trace over 10^5 traces. (d) A similar analysis to part (c), with 1000 copies of each species as opposed to 100. The rate constants are $kAM = 1$, $kNoise = 1$, and $kStimulus = 0.1$, and the probabilities are calculated with one decision cycle per trace over 10^5 traces. As before, $\text{WY} = \text{WZ} = (1000 - \text{WX})/2$.

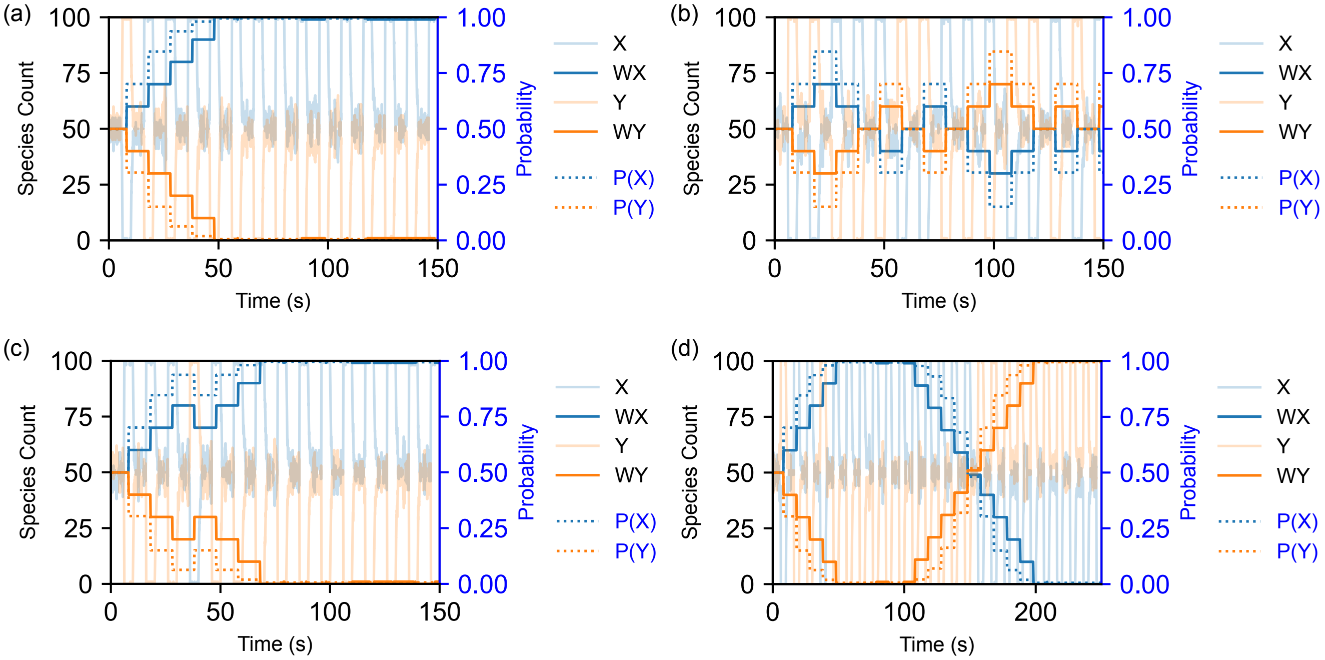

Fig D: Operant conditioning in deterministic environments. (a) Representative timecourse for an operant conditioning simulation in which the environment responds to decision X with 10 Good molecules and to decision Y with 10 Bad molecules. The system is initialized with 50 copies of each weight species (WX and WY) and 15 stimulus/relaxation cycles are performed. Rate constant values are $kBias = 30$, $kAM = 1$, $kNoise = 1$, $kFB = 1$, and $kFBDeg = 1$. Species counts for X, Y, WX, and WY are plotted on the left-hand axis. The corresponding decision probabilities $P(\text{X})$ and $P(\text{Y})$ are plotted on the right-hand axis; these are calculated by using the "kBias = 30" transfer curve from Figure 2 of the main text as a calibration to convert weight species counts into the corresponding probabilities. This pattern of guaranteed positive feedback for decision X and guaranteed negative feedback for decision Y drives the system inexorably into the state where $P(\text{X}) = 1.0$. (b) Representative timecourse for an operant conditioning simulation in which the environment responds to both decisions (X and Y) with 10 Bad molecules. The system is initialized with 50 copies of each weight species (WX and WY) and 15 stimulus/relaxation cycles are performed. Rate constant values and plotting are as in part (a). This environment causes the decision probabilities to fluctuate around $P(\text{X}) = P(\text{Y}) = 0.5$, as the system receives negative feedback regardless of which decision it takes. (c) Representative timecourse for an operant conditioning simulation in which the environment responds to both decisions (X and Y) with 10 Good molecules. The system is initialized with 50 copies of each weight species (WX and WY) and 15 stimulus/relaxation cycles are performed. Rate constant values and plotting are as in part (a). This environment drives the system to either $P(\text{X}) = 1.0$ or $P(\text{Y}) = 1.0$, depending on which choice happened to be favored initially, as the system receives positive feedback regardless of which decision it takes. (d) Representative timecourse for an operant conditioning simulation in which the environment initially responds to decision X with 10 Good molecules and to decision Y with 10 Bad molecules. At time $t = 100$, the Good and Bad feedback responses are switched. The system is initialized with 50 copies of each weight species (WX and WY) and 15 stimulus/relaxation cycles are performed. Rate constant values and plotting are as in part (a). This environment drives the system to the $P(\text{X}) = 1.0$ state initially, then drives it back to the opposite $P(\text{Y}) = 1.0$ state when the feedback polarity switches. This demonstrates the ability of operant conditioning systems to adapt to changing environmental conditions.

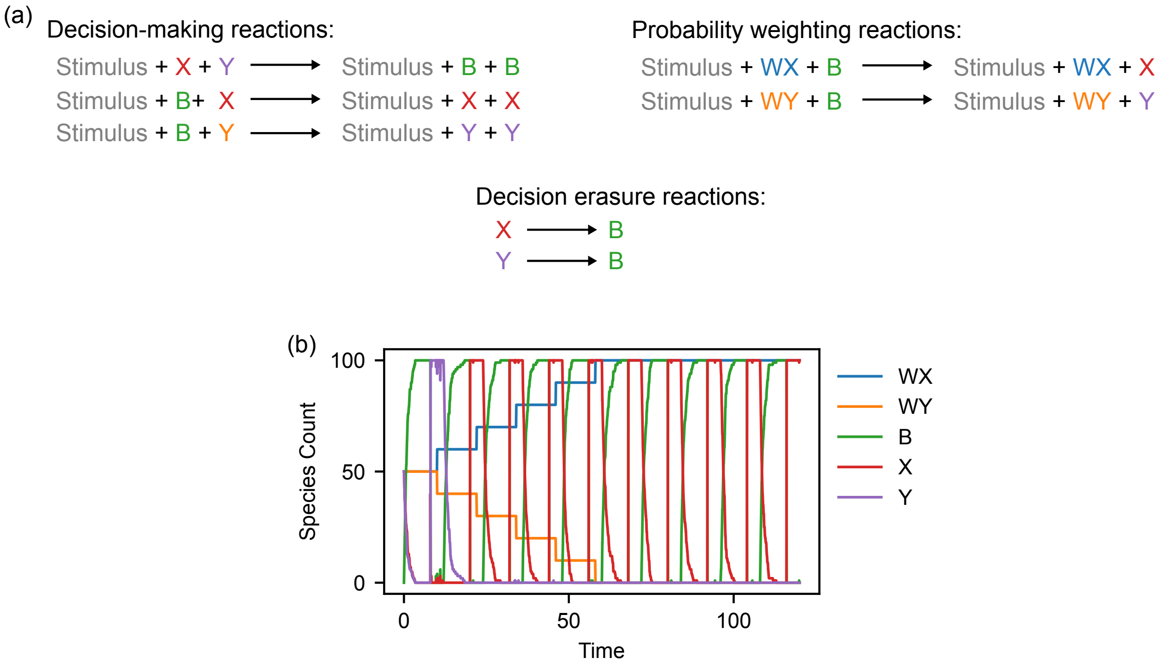

Fig E: An alternate module to replace entropy generation for probabilistic decision-making. (a) Chemical reaction network for a simple decision-making system using the alternative decision erasure system. In this scheme, the AM reactions are as before, the probability weighting reactions are also catalyzed by the Stimulus species, so that the steady state of the system in the absence of Stimulus is a population consisting entirely of the intermediate species. Finally, the decision erasure reactions cause the X and Y decision species to degrade into B whenever the Stimulus is removed. This erases the preceding decision and sets up the system for the next conditioning cycle. The rate constants are *kAM* the decision-making reactions, *kBias* for the probability weighting reactions, and *kNoise* for the decision erasure reactions. (b) Representative simulation demonstrating conditioning of the decision erasure system. In this simulation, the environment provides deterministic feedback consisting of 10 Good molecules for the X state and 10 Bad molecules for the Y state. Rate constant values in this simulation are $kNoise = kAM = 1$ and $kBias = 3$. There are 10 cycles shown; each 8 time units without Stimulus present and 4 time units with. Of note is the fact that the system resets itself into an indeterminate state via the decision erasure reactions, producing a stable state with 100 copies of the B species present at the end of each conditioning cycle. This simulation shows that the decision erasure system can be conditioned in a similar manner to the system based on reversible entropy generation reactions that was studied extensively in the main text.
